# IL-13 expression by blood T cells and not eosinophils is increased in asthma compared to non-asthmatic eosinophilic bronchitis

**DOI:** 10.1186/1471-2466-9-34

**Published:** 2009-07-14

**Authors:** Salman Siddiqui, Glenn Cruse, Susan Mckenna, William Monteiro, Vijay Mistry, Andrew Wardlaw, Christopher Brightling

**Affiliations:** 1 Institute of Lung Health, University of Leicester, Leicester, UK

## Abstract

**Background:**

In asthma interleukin (IL)-13 is increased in the airway compared with non-asthmatic eosinophilic bronchitis. Whether this differential expression is specific to the airway or is more generalised is uncertain.

**Methods:**

We sought to examine IL-13 expression in peripheral blood T-cells and eosinophils in asthma and non-asthmatic eosinophilic bronchitis. Peripheral blood CD3+ cell and eosinophil intracellular IL-13 expression from subjects with asthma, non-asthmatic eosinophilic bronchitis and healthy controls was assessed. The effect of priming by asthmatic serum on the release of IL-13 by peripheral blood mononuclear cells from healthy subjects was examined and the serum from these subjects was analysed for a range of chemokines and cytokines.

**Results:**

The median (IQR)% intracellular IL-13 expression by CD3+ cells was increased in asthma [5.3 (2.7–9.8)%; n = 12] compared to non-asthmatic eosinophilic bronchitis [1.1 (0.5–3)%; n = 7] and healthy controls [1.7 (0.2–3%); n = 9] (p = 0.02), but was not significantly different in eosinophils across the groups. IL-13 released from healthy peripheral blood mononuclear cells (n = 10) was increased by asthmatic serum [117 (47.8–198)pg/ml] compared to control [78.5 (42.6–128)pg/ml; p = 0.02), but was not affected by non-asthmatic serum.

**Conclusion:**

Our findings support the view that IL-13 expression is increased in peripheral blood-derived T cells in asthma and that asthmatic serum up-regulates IL-13 release from healthy peripheral blood mononuclear cells.

## Background

Asthma is characterised by the presence of variable airflow obstruction, airway hyper-responsiveness (AHR), and an airway inflammatory response often characterised by Th2-mediated eosinophilic airway inflammation [1] with mast cell infiltration of the airway smooth muscle (ASM) bundle[2]. The Th2 cytokine interleukin (IL)-13 has been implicated in the pathogenesis of asthma[3]. Its central role in the asthma paradigm is supported by human studies that have reported increased IL-13 mRNA expression in bronchial biopsies from subjects with moderate asthma[4,5] and from sputum cells from corticosteroid naïve and inhaled corticosteroid treated asthmatics[6]. In addition, following allergen challenge in mild asthmatics bronchoalveolar lavage IL-13 concentration was upregulated[7]. This association between IL-13 and asthma in humans is supported by animal models[8]. T-lymphocyte deficient mice have shown exogenous addition of IL-13 promotes AHR and airway inflammation, while neutralisation of IL-13 in murine models can resolve these features[9].

Importantly comparisons between asthma and non-asthmatic eosinophilic bronchitis (EB), a common cause of chronic cough[10] in which disordered airway physiology is not a feature, have been informative about the key immunopathological features of asthma. Over expression of IL-13 in sputum [11-13], bronchial submucosa[11,12] and co-localisation to mast cells in the ASM-bundle[14] are features of asthma that are not shared by EB and have therefore further supported its role in the pathogenesis of AHR. Whether this differential IL-13 expression is specific to the airway or is a generalised phenomenon present in peripheral blood cells is uncertain.

We hypothesised that IL-13 expression is upregulated in peripheral blood T-cells and eosinophils in asthma compared to EB and healthy controls. We have further considered the possibility that asthmatic serum contains a pro-inflammatory stimulus that primes IL-13 release from healthy subjects.

## Methods

### Subjects

Subjects with asthma (n = 24) and EB (n = 7) and healthy volunteers (n = 16) were recruited from Glenfield Hospital outpatients, staff and by local advertising. All subjects were non-smokers with a smoking history of < 10 pack years; had been free of exacerbations and on stable treatment for 8 weeks prior to entry into the study. EB was defined according to the American College Of Chest Physicians (ACCP) criteria[15]. Asthma was defined by one or more of the following objective criteria: significant bronchodilator reversibility of > 200 mls, a provocation concentration of methacholine causing a 20% fall in FEV_1 _(PC_20_) of less than 8 mg/ml or a peak flow amplitude % mean over 2 weeks of more than 20%. Severity was classified using the current global initiative for asthma (GINA) treatment steps[16]. Normal subjects had no history of respiratory disease and normal spirometry. The Leicestershire ethics committee approved the study and all patients gave their written informed consent.

### Protocol and clinical characterisation

Subjects underwent spirometry, allergen skin prick tests for *Dermatophagoides pteronyssinus*, dog, cat and grass pollen, measurement of exhaled nitric oxide (eNO) concentration (measured at 50 mls/s NIOX; Aerocrine, Stockholm, Sweden), a methacholine inhalation test[17] and sputum induction[18].

### Isolation of peripheral blood mononuclear cells (PBMC) and eosinophils

Mononuclear cells and eosinophils were isolated from peripheral blood. Briefly, 100 mls of heparinised peripheral blood was venesected from a subgroup of the patients with asthma (n = 12), EB (n = 7) and healthy controls (n = 9). After an initial step of Dextran red cell sedimentation, the plasma layer was removed and after centrifugation the cell pellet was resuspended over Histopaque 1077 (Sigma, Poole, Dorset, UK). After further centrifugation the mononuclear cell interface was collected and washed twice with phosphate buffered saline. For eosinophil separation the remaining pellet after histopaque centrifugation underwent red cell lysis followed by negative immunomagnetic selection using anti-CD16 coated immunomagnetic beads. There were insufficient eosinophils isolated to undertake further experiments from 3 healthy controls and 1 subject with EB. Cell purity and viability was assessed and was > 95% in all subjects. Following purification PBMC and eosinophils were cultured at 1 × 10^6 ^cells/ml in RPMI 1640 medium supplemented with 10% fetal calf serum and either left unstimulated in the presence of brefeldin or stimulated with phorpbol myristate acetate (PMA) [5 ng/ml; Sigma Chemical Co, UK] and calcium ionophore [250 ng/ml; Sigma, UK] in the presence of the fungal protein brefeldin [10 μg/ml] for 4 h at 37°C.

### Intracellular Flow cytometry for IL-13

The cells were fixed in 4% paraformaldehyde (Sigma, UK) and stored overnight at 4°C in PBS, 0.5% BSA. The following day the cells were washed and incubated with CD3-RPE (mononuclear cells only) (BD biosciences, UK) permeabilised in 4% paraformaldehyde, 0.1% saponin (Sigma, UK) for 15 min on ice and labelled with IL-13-FITC (BD biosciences, UK) or isotype controls and analysed by two-colour flow-cytometry as described previously[19].

### Stimulation of healthy PBMC with asthmatic serum

PBMC from 10 healthy controls, including 3 subjects that had participated in the first part of this study, were stimulated with pooled serum from asthmatic subjects and PBMC from 4 of these subjects were stimulated with pooled serum from non-asthmatics.

PBMC stimulation was carried out in 24-well cell culture plates in duplicates in the presence and absence of 0.1, 1 and 10% human serum. IL-17E has been implicated to promote IL-13 release from PBMC and may be an important mediator in the asthmatic serum. Therefore the serum stimulation was undertaken with and without recombinant human IL-17E (100 ng/ml) and or IL-17E neutralising antibody (20 μg/ml) (R&D Systems, Abingdon, Oxford, UK). 500 μl of PBMC suspension was added to each well equating to 2 × 10^6 ^PBMC/well. Finally, 250 μl of either DMEM alone, or DMEM containing 4 × final concentration (final concentration was 20 ng/ml) of PMA (Sigma, UK) was added to the cells. The cells were incubated for 16 h at 37°C in a humidified atmosphere flushed with 5% CO_2_. Following the incubation, PBMC were decanted into 1.5 ml Eppendorf^® ^tubes and centrifuged at 400 × g for 5 min. The supernatants were removed and stored at -20°C for analysis.

IL-13 was measured using a sandwich ELISA (Bender MedSystems, UK). The limit of detection of the ELISA was 1.56 pg/ml. The IL-13 concentration in the pooled asthmatic and non-asthmatic serum was subtracted from the concentration of IL-13 released by stimulated PBMC primed with serum.

### Mesoscale analysis of serum from asthmatics and healthy controls

The concentration of a panel of cytokines and chemokines were measured in serum from the 10 healthy subjects that had participated in asthmatic serum-stimulated PBMC experiments and 12 asthmatics using the mesoscale discovery system. This assay uses electrochemiluminescence detection and pattern arrays (Mesoscale Discovery, Gaithersburg, Maryland, USA). The panel included the cytokines (IFN-γ, IL-1β, IL-2, IL-4, IL-5, IL-8 (CXCL8), IL-10, IL-12p70, IL-13, TNF-α) and chemokines (CCL11, CCL4, CCL26, CCL17, CXCL10, CCL2, CCL22, CCL13). The limits of detection of the mesoscale system were, [CCL2; 1 pg/ml], [CCL4; 20 pg/ml], [CCL11; 10 pg/ml], [CCL13; 100 pg/ml], [CCL17; 50 pg/ml], [CCL22; 150 pg/ml], [CCL26; 200 pg/ml], [CXCL8; 1 pg/ml], and [CXCL10; 200 pg/ml], all other limits of detection were 1 pg/ml.

### Statistical analysis

Subject characteristics were described using descriptive statistics. Intracellular IL-13 was expressed as a percentage above isotype control and expressed as the median and interquartile range (IQR). Comparison across the three groups was performed using a Kruskal-Wallis one-way analysis of variance with Dunns post test for inter-group comparisons. Paired data were analysed using the Wilcoxon matched pairs t-test.

## Results

Baseline clinical characteristics are shown in Table 1. The subjects with severe asthma and non-asthmatic eosinophilic bronchitis were older than those with mild asthma or healthy controls. Eosinophilic inflammation was a feature of subjects with asthma and non-asthmatic eosinophilic bronchitis, whereas airway hyper-responsiveness was reserved to those with asthma.

**Table 1:** Clinical Characteristics

	**Control** **(n = 16)**	**Asthma Mild-moderate** **(GINA 1 = 6, GINA 2–3 = 6)**	**Asthma Severe** **(GINA 4 = 6, GINA 5 = 6)**	**Eosinophilic Bronchitis** **(n = 7)**
**Age (yrs)~**	32.2 (2.3)	37.3 (3.7)	51.0 (4.3)*	53.6 (6.2)*
**Sex M:F**	7:9	7:5	4:8	6:1
**Atopy (%)**	46	83**	67**	43
**Disease Duration (yrs)**	N/A	22.0 (3.8)**	30.4 (4.9)**	8.4(1.8)
**BDP Equivalent (mcg)**	N/A	355 (132)	1633 (175)	1149(420)
**Oral Prednisolone** **(mg/24 hrs)**	N/A	N/A	5.6 (2.0)	0
**ENO (50)** **(Ppb) #**	18 [9–37]	28 [19–46]	19 [14–25]	34 [18–64]
**PC_20 _(mg/ml)#**	>16	1.4 [0.4–4.9]**	0.45 [0.1–1.7]**	>16
**FEV_1 _% predicted~**	109 (3.3)	90 (8.7)	78 (6.0)*	95.7(5.6)
**FEV_1_/FVC~**	86.3(1.7)	79 (3.4)	72 (3.5)*	80.0(4.4)
**Induced sputum**				
**Eosinophils (%)#**	0.7 [0.2–2.7]	2.2 [0.4–13.0]*	1.9 [0.8–4.6]*	4.6 [1.3–16.5]*
**Neutrophils (%)~**	43 (12.6)	46 (9.9)	66.5 (7.1)	43.5(5.6)

~ mean (SEM), # geometric mean (95% confidence interval), N/A not applicable

*p < 0.05 vs. control, **p < 0.05 vs. control and/or EB

### IL-13 expression is increased in PBMC but not eosinophils in asthma compared to EB

**Figure 1:**
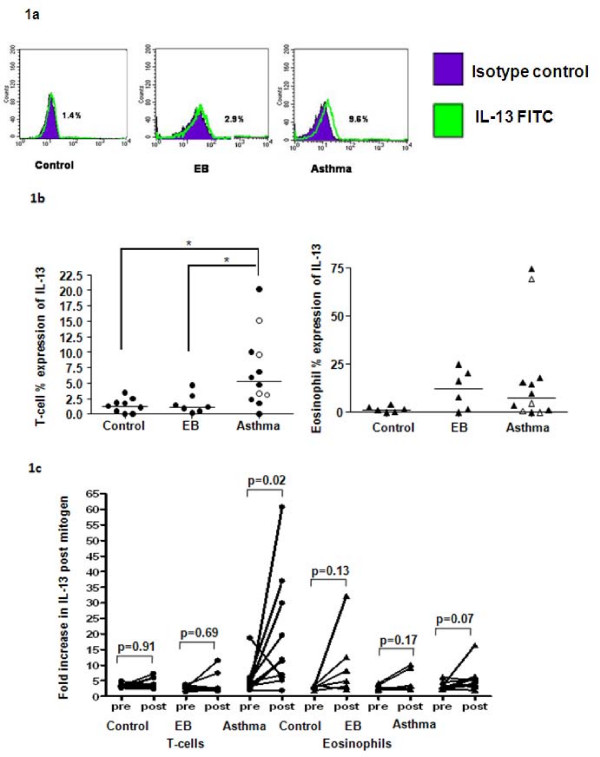
**Intracellular expression of IL-13 in CD3+ T cells**. **1a**: Representative histograms and dot plot of the percentage of CD3+ T cells expressing IL-13 in asthma, EB and healthy controls at baseline. Solid peak (isotype control), solid line (IL-13 FITC conjugate). **1b**: Increased % expression by CD3 T cells of IL-13 in asthma at baseline compared to patients with EB and healthy subjects. *p < 0.05 (asthma subdivided into those with mild-moderate disease closed circles and severe asthma open circles). **1c**: Upregulation of CD3+ T cell and eosinophil IL-13 expression in asthma after mitogen stimulation. Fold increase in IL-13 fluorescence compared to isotype control before and after mitogen stimulation.

Intracellular expression of IL-13 was significantly increased at baseline in peripheral blood CD3+ T cells in asthma compared to patients with EB and healthy controls (figures 1a &1b). The median (IQR) % intracellular expression was 5.3(2.7–9.8)% asthma, 1.3(0.2–2.1)% EB and 1.1(0.2–3%) controls (p = 0.007 Kruskal-Wallis; p < 0.05 Dunn's post-hoc test asthma versus EB/Controls). Following stimulation there was a significant increase in the expression of IL-13 in CD3+ T cells in asthma (p = 0.02), but not in patients with EB or healthy controls (figure 1c). In asthma, there was no correlation between the T-cell intracellular IL-13 expression and the sputum eosinophil count or AHR (r = 0.17; p = 0.69 and r = 0.26; p = 0.46 respectively) and no difference between the intensity of expression in those with or without atopy (p = 0.11).

The median (IQR) baseline % intracellular expression of IL-13 in eosinophils was not significantly different between subjects with asthma 7.2 (0–16.7)%, EB 12.1 (0–22.6)%, or healthy controls 1.4 (0–3.3)% (p = 0.2 Kruskal Wallis; figure 1b). There was no significant increase in IL-13 expression by eosinophils following stimulation (figure 1c).

### Stimulation of PBMC from healthy subjects with pooled asthmatic serum leads to increased IL-13 release that is not mediated by IL-17E

**Figure 2:**
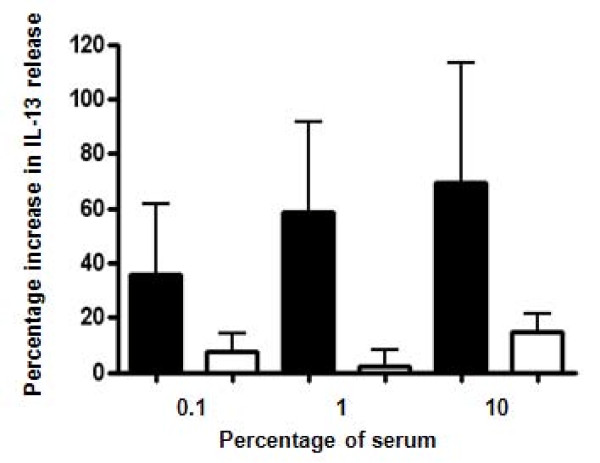
**Asthmatic serum increases IL-13 release from healthy T Cells**. Mean (SEM) percentage increase in IL-13 release by PBMC from healthy donors after stimulation with mitogen in the presence of 0.1–10% pooled serum from subjects with asthma and non-asthmatic controls. Closed bars PBMC (n = 10) stimulated with asthmatic serum, open bars PBMC (n = 4) stimulated with non-asthmatic serum.

The median (interquartile range) IL-13 release from PBMC stimulated with PMA was 78.5(42.6–128) pg/ml]. This was significantly increased after stimulation with 1% asthmatic serum (117 [47.8–198] pg/ml; p = 0.027; mean [SEM]% increase 36 [27]%); with a non-significant trend towards an increase with 10% serum (90.2 [47.7–278.8] pg/ml; p = 0.08; 58 [32]% increase), but not 0.1% serum (85 [48.9–197.2] pg/ml; p = 0.4; 70 [43]% increase). The PMA-mediated IL-13 release by PBMC was not affected by stimulation with serum from non-asthmatic individuals (figure 2).

IL-13 release was not altered by recombinant IL-17E [79.5(62.6–170.2) pg/ml]; p = 0.2 or IL-17E neutralising antibody (data not shown).

### Systemic inflammation in severe asthma

**Figure 3:**
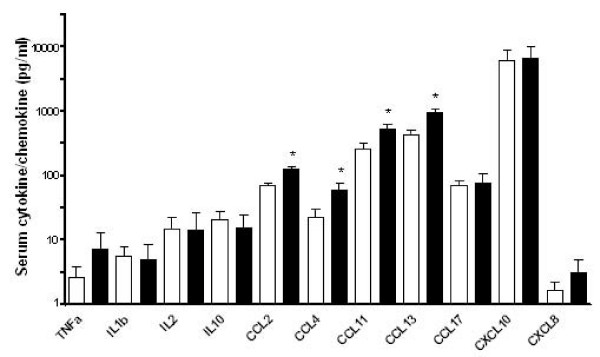
**Cytokine and chemokine concentrations in serum from asthmatics and healthy controls**. The concentration of a panel of cytokines and chemokines in serum from asthmatics (closed bars) and healthy controls (open bars). *p < 0.05.

We found that the following chemokines were significantly up-regulated in the serum in asthma compared to healthy subjects CCL2, CCL4, CCL11 and CCL13 (p < 0.05). In contrast we did not find increased cytokine expression in asthmatic serum (Figure 3).

## Discussion

We have shown that in asthma IL-13 expression is increased in peripheral blood T cells, but not eosinophils compared to subjects with EB or healthy subjects. In addition IL-13 released by PBMC from healthy controls was increased by asthmatic serum. This suggests that the increased IL-13 expression in asthma may be mediated by a hitherto undetermined pro-inflammatory stimulus in asthmatic serum. This stimulus is not IL-17E, and we were unable to find a plausible candidate mediator with mesoscale analysis of a range of cytokines and chemokines.

We report here for the first time differential expression of intracellular IL-13 in peripheral blood T-cells from asthmatics compared to subjects with EB and healthy controls. This supports our hypothesis that increased IL-13 expression in asthma is not reserved to the airway but is also present in peripheral blood inflammatory cells. Our findings are consistent with a previous study by *Park et al*[20], in which IL-13 secretion by PBMC was increased in asthma compared to EB and healthy controls. In addition we have shown for the first time that IL-13 expression in eosinophils was not different between subjects with asthma and EB, although there was a non-significant trend towards increased expression in eosinophils in the disease groups compared to controls. This suggests that the observed up-regulation of the IL-13 axis by airway eosinophils in asthma [11-13] may be influenced by the local microenvironment.

Importantly, we have demonstrated that serum from asthmatics but not from non-asthmatics can promote increased IL-13 release from healthy PBMC suggesting that asthmatic serum contains an activator of the IL-13 pathway. We investigated the role of IL-17E a known pro-inflammatory primer for IL-13 release as a potential candidate[21], but our findings do not support a role for IL-17E as a primer for IL-13 release in peripheral blood. In addition we were unable to identify a potential pro-inflammatory candidate in serum across a range of chemokines and cytokines assessed by mesoscale analysis. Therefore further work is required to assess the potential role of systemic inflammation in mediating up-regulated Th2 cytokine expression in asthma.

Our findings further strengthen the argument for IL-13 being a key mediator in the pathogenesis of AHR in asthma. IL-13 may promote AHR by a variety of diverse mechanisms. In vitro IL-13, but not IL-4, has been shown to attenuate ASM relaxation to β-agonists[22] and augment contractility to acetylcholine[23] suggesting that IL-13 may induce AHR by directly activating ASM. Indirectly IL-13 may modulate AHR by its effects on airway smooth muscle proliferation[24] and interactions with the airway epithelium[25]. In contrast to *Park et al *[20], we were unable to demonstrate a correlation between AHR and T-cell IL-13 expression suggesting that the relationship between the intensity of IL-13 expression and AHR is complex as we have previously described in severe asthma[12].

One potential criticism of our study is its cross-sectional design as this does limit our interpretation of the possible effects of corticosteroid therapy and the longitudinal variability of IL-13 expression in peripheral blood cells. However, we are confident that we have established key differences in IL-13 expression between asthma and EB by peripheral blood T-cells supporting the view that the differences observed in the airway and also present systemically. Further work is required to explore the mechanisms driving this differential IL-13 expression.

## Conclusion

In conclusion we have found that IL-13 expression is increased in peripheral blood T-cells in asthma compared to EB and healthy subjects, which in part may be due to a pro-inflammatory stimulus in asthmatic serum. This further supports the role of IL-13 in asthma.

## Competing interests

CB has received consultancy fees and research funding from GlaxoSmithKline, MedImmune, and AstraZeneca. AW has received research funding from GlaxoSmithKline and AstraZeneca and Wyeth. All other authors have no conflict of interest.

## Authors' contributions

SS undertook the laboratory experiments with assistance from GC, WM, and VM and the patient characterisation with SM and WM. CB and AW supervised the laboratory work. CB was the principal investigator for the study and wrote the manuscript with SS. All authors read and approved the final manuscript.

## Pre-publication history

The pre-publication history for this paper can be accessed here:


